# Cynanche Laryngea Chronica, or Chronic Laryngitis

**Published:** 1838-01-17

**Authors:** N. Chapman

**Affiliations:** Professor of the theory and practice of Physic, in the University of Pennsylvania


### MEDICAL EXAMINER.

DEVOTED TO MEDICINE, SURGERY AND THE COLLATERAL SCIENCES.

**EDITED by J. B. BIDDLE, M. D. and M. CLYMER, M. D.**

No. 2. PHILADELPHIA, WEDNESDAY, JANUARY 17, 1838. Vol. I.

### CYNANCHE LARYNGEA CHRONICA,

### OR CHRONIC LARYNGITIS.

By N. Chapman, M. D., *Professor of the theory and practice of Physic, in the University of Pennsylvania.*

(CONCLUDED FROM PAGE 5.)

Enough, perhaps, has been disclosed in the preceding reviews, to indicate the diversity of treatment appropriate to the several forms, or states of the disease. But since it is not easy to ascertain the exact general pathological nature of the case, and still less the condition of parts mainly concerned; our practice, I apprehend, is seldom guided by enlightened discrimination, or any very definite principles. It is obviously required to the attainment of success, that in engaging in a case, the first endeavour should be the determination of these points, as accurately as possible.

Being persuaded that the case originates in some peculiar cause, as the elongation of the uvula, or repulsion of an eruption, or of the existence of either of the diatheses, to which allusion has been made; the practice of course must be, in some degree, specially directed by such views, and regulated according to the circumstances of the moment. It were foreign to the occasion, to make these considerations at present the subject of further discussion, they coming in with greater propriety hereafter, under the history of the diseases, phthisis, scrofula, syphilis, &c., to which they immediately belong. My remarks will hence be confined principally to the management of chronic inflammation of the larynx, of the ordinary kind, in a sound constitution, which may, perhaps, be deemed the only case where efforts of cure are much encouraged.

As might be supposed, the remedies to meet the indications here, are bleeding, general and local, blisters, or other counter irritants, frictions with the emetic tartar ointment, or the croton oil, till pustulation is induced, and setons, issues, &c. Great advantage also is derived from the application, once or twice a day, of burnt alum over the entire surface of the fauces, which may be done with a small brush. It is equally proper, whether there be inflammation or not in these parts, unless it is active or intense. The mode of its operation seems to be, by setting up a new action, followed by a copious secretion of thin fluids, which extending to the larynx, subverts the one existing in its mucous lining.

By this plan perseveringly pursued, relief is undoubtedly sometimes procured. But it very often fails; and occasionally, owing to circumstances not always appreciable, the depletory part of it, urged to any extent, proves positively detrimental, by inducing weakness, without abating the force of the disease, or in any way making a favourable impression. In such an event, we are left nearly destitute of resources; for an opposite course, comprehending the medicinal tonics and other means of invigoration, are even more pernicious, raising up a degree of excitement that hastens with fearful rapidity the catastrophe. Treatment of this kind is here totally misapplied, and is only adapted to cases arising out, or otherwise associated with, a feeble or cachectic state of system.

On the reduction of the activity of the phlogosis, no inconsiderable confidence has been reposed in an alterative course of mercury, with some of the narcotics, hemlock, henbane, or opium, and ipecacuanha. But I confess that my trials of these articles have mostly ended in disappointment, though I still think that they are not to be neglected, and particularly mercury. Holding it to be nearly infallible, a late writer, of good repute, declares, " that in every case not depending on, or connected with disorganization of structure, it will, probably, afford relief, no matter at what period of the disease it is administered, or under what unpromising circumstances."* He orders it in small or large doses, as may seem most proper to effect salivation, which he supposes essentially necessary to the cure—and in some urgent cases has given as much as ten grains of calomel, four times a day. As soon, continues he, " as its specific influence becomes developed, the disease begins to decline, and it seldom requires more than a week or ten days to render the cure complete.

It were well if this account, with ample allowance for the exaggeration of the enthusiasm of the moment, could be verified. Experience, however, has exposed its fallacy, and it affords another melancholy instance how little reliance is sometimes to be placed on medical testimony, owing to hasty and crude conclusions. Long ago mercury was

*Porter on the Diseases of the Larynx and Trachea.

tried in this city, in every mode, as an alterative and salivant, so often with manifest harm, though now and then successfully, that under the impression of its proving, on the whole, mischievous, the use of it now is nearly abandoned. But we shall presently see, that this is turning from the one to the opposite extreme. As inculcated by the authority just cited, the practice is singularly wanting in discrimination, and partakes somewhat of the character of empiricism. Granting its applicability to certain cases, which he has not adequately pointed out, surely it would be contra-indicated, wherever a tuburcular or mercurial diathesis exists, the best evidence concurring to shew its baneful influence, in either of these conditions.

It may safely be affirmed, that with the exceptions of perhaps some of the syphilitic cases, and those growing out of certain derangements of the digestive apparatus, the only state in which it is admissible, is that of ordinary inflammation, independent of any general depravity of system, and even under the most favourable circumstances, its use is to be regulated with great circumspection. Thus appropriately administered, I have witnessed in several cases, the most unequivocal advantage from it.

Emetics, at one time proposed, are now generally confessed to be prejudicial, though I suspect this unfavourable estimate of them has not been very wisely formed. Exactly as they may be resorted to with discrimination, or otherwise, so will they prove beneficial or the reverse. Conspicuously injurious at an advanced period of the disease, they sometimes prove of the greatest service in the forming or incohative stage of it, and especially in those cases blended with considerable affection of the fauces and their immediate connections. As already intimated, it is here that the stomach is essentially in fault, and vomiting occasionally repeated, I have known to prove very successful, even to the accomplishment of perfect cures.

Not much is to be gained from expectorants. Led by analogy, the balsams particularly, have been thought to promise fairly, and though this *a priori* recommendation of them, is sustained by the unqualified testimony of some writers, I can attach only a very limited value to them from my own observation. There is, indeed, a plain reason for their general failure in this case. The disease is, for the most part, deeply implanted in the subcellular texture, where such articles are without power, they doing good only, when the mucous lining is implicated, either ulcerated or pouring out inordinate secretions, over which and especially the latter, they have a restraint unquestionably.

Nothing would be more unprofitable than to enumerate the means which have been applied to the cure of the disease in the advanced stages. Connected with great vitiation of system, there are then usually ulcerations of the larynx; of the remedies, perhaps those now mostly employed, are the compound syrup of sarsaparilla, and the inhalation of certain balsamic and vulnerary vapours, as of the tolu, rosin, tar, &c. Excepting under the circumstances which have been defined, of the probable utility of the same articles when given internally, they must be nugatory in this mode of administration. Generally indeed, they prove so irritating, that they cannot be endured.

From some recent trials I have made, I am inclined to believe that the fumes of iodine and chlorine are deserving of more attention, though still mere palliatives. The only cure I have accomplished by such means was by mercurial inhalations. It is, however, true, that in the very mildest form of the affection, dependent probably on mere relaxation of parts, the vapours of Hoffman's liquor and laudanum mixed, or of a digest of ether and cicuta, I have known sometimes to operate very beneficially.

Œdema of the larynx existing, topical bleeding and vesication are usually directed to exterminate the remnant of inflammation, if any is suspected to continue, and to promote the absorption of the effused fluid, frictions with the mercurial or iodine ointment are employed. But so far as I have seen, no benefit accrues from these applications, nor indeed from anything else. It is here that I would again suggest the evacuation of the fluid by incisions.*

Distrustful entirely of medical treatment, I have, for some time, restricted my advice to whatever conduces most to the comfort of the individual, instead of harassing him by unavailing efforts of cure in this and other hopeless states of the disease. An exception however may, perhaps, be found to this sweeping condemnation of active practice. Laryngotomy has been advised, and even performed under these apparently desperate circumstances, and with such success as to merit, perhaps, further trials. There is a case reported by Sir Charles Bell, when life was protracted by it for seven weeks, and which probably might have done well, had not the cure been frustrated by an untoward occurrence. In two other instances reported by Drs. Porter and Marshall Hall, an entire recovery took place, and Professor Regnoli has been no less furtunate in two cases. Mr. Liston has also succeeded in one, and Mr. Cullen, a Surgeon of Edinburgh, more recently in another.

That additional proof of the efficacy of the operation might be collected by further researches is not unlikely.

Nevertheless, no very sanguine expectations can reasonably be entertained from this resource as a general means of cure. The larynx itself, as we have seen, may be irreparably affected by various organic lesions, or where these do not exist, the trachea or the lungs are so deeply engaged in the diseased action, that no local expedient of this kind can possibly avail. It was owing to these insuperable impediments that Mr. Lawrence twice failed in the operation—finding in the one instance, the membrane so thickened and granulated, as nearly to close the rima glottidis, and in the other, the lungs much disordered.

Easy were it to multiply proofs of the inadequacy of the operation, though cases of failure are much less apt to be proclaimed than of success. Examples however, have occasionally occurred of the disease, without any serious structural derangements of the windpipe, or of the lungs, and it is to

*This was recommended in a previous lecture on acute Laryngea Œdematosa.

such only that the operation is applicable with any certainty of advantage. Could these cases be accurately determined there ought, perhaps, to be little hesitation in resorting to it, provided life were endangered by the disease. But the diagnosis from symptoms, is very obscure, and I am afraid we shall very generally be not a little embarrassed in coming to a just decision.

It is true, that by percussion and auscultation, information might be derived of the state of the lungs, whether they are implicated or not, and probably as to the kind and degree of implication. No light, however, is shed from these sources in relation to the condition of the larynx or trachea, and we shall be left to proceed here on conjectural inferences for the most part. This is a question on which we must be controlled mainly by the circumstances of each case, to be determined in a great degree by the medical attendant himself, and I shall not discuss it further. On the whole, in suggesting the operation, I have been influenced not so much by any real appreciation of it, as by the old maxim :

"Anceps remedium, melius est, quam nullum."

Not much have I to say in regard to the regimen in this disease. As to diet, it is to be accommodated to the diversions which it presents in its essential character, as well as its several stages. Enough may it be to state on this point, that so long as any activity of phlogosis remains, it must be low and abstemious, consisting of the farinaceous and other light and digestable vegetable matter—and, under other circumstances, to be more generous and nutritious—though always excluding whatever of food or drink that has a stimulating or heating tendency.

During the continuance of the same phlogistic condition, a state of repose ought to be observed, and on its subsidence, exercise freely taken on foot, or in a carriage, or on horseback, whenever the weather admits of it.

Every precaution should be practiced in the avoidance of catching cold, and with this view the individual is to be protected by adequate clothing, and cautioned against hazardous exposures.

Not less important is it, that he refrains from unnecessary exercise of the voice, even in conversation, speaking in a low tone and slowly ; and if he belong to a profession in which it is to be exerted, instantly to abandon it.

Much may be expected, early in the disease, by a removal to a mild equable climate, and nothing in the advanced stages. But where this cannot be accomplished, confinement in a room of a regulated temperature, in cold or damp, austere weather, will prove the best substitute, and must be enforced.

